# Characterization of Pseudomonas strains from sinusitis patients

**DOI:** 10.1186/1471-2334-12-S1-P64

**Published:** 2012-05-04

**Authors:** Shanmugam Sasikala, Thangiah Sundararaj

**Affiliations:** 1 Department of Microbiology, Dr. ALMPGIBMS, University of Madras, Chennai, India; 2 Department of Microbiology & Biotechnology, Presidency College, Chennai, India

## Background

Sinusitis affects significant percentage of population causing considerable long term morbidity. *P.aeruginosa* is responsible for about 13% of the sinusitis cases. *Pseudomonas* produces hemolysin, rhamnolipid, a membrane glycolipid, secretory pigments like pyocyanin and 1-hydrophenazine which can reduce ciliary beating and mucociliary clearance. Alginate functions as an adhesion, thereby preventing phagocytosis.

## Methods

This study involved the characterization of 22 *Pseudomonas* strains isolated from endoscopic pus of 170 sinusitis patients. Tests for the production of haemolysin, protease, lipase, lecithinase, slime, leucocidin toxin were performed. Cell adherence property was also evaluated. Antibiotic susceptibility tests were performed.

## Results

All the strains produced β hemolysin. Coloured pigments ranging from bluish green to green, pink and purple were produced. Among the 22 strains, 18 were positive for proteases, 17 for lipases and 3 for lecithinase. All the 22 isolates produced leucocidin toxin, which killed all the leucocytes in 10 minutes. All the strains produced slime layers ranging from 20-35 mm height. The rate of cell adherence to the nasal epithelial cells depended on both the clinical strains and the individuals from whom nasal epithelial cells were taken. Clinical strains attached more efficiently (p=0.001) than the environmental strains. All the strains were sensitive to Amikacin, Gatifloxacin, Norfloxacin and Ofloxacin. To the antibiotics, Ceftrioxzone, Gentamicin, Cefotaxime, Chloramphenicol and Piperacillin, 18%, 9%, 28%, 59% and 59% of the strains showed resistance respectively.

## Conclusion

Thus it is evident that *P.aeruginosa* produces virulence factors, demonstrates resistance towards multiple antibiotics. Successsful treatment necessitates a thorough knowledge of the prevailing bacteria.

